# Sequence exploration reveals information bias among molecular markers used in phylogenetic reconstruction for *Colletotrichum* species

**DOI:** 10.1186/2193-1801-3-614

**Published:** 2014-10-17

**Authors:** Sephra N Rampersad, Fazeeda N Hosein, Christine VF Carrington

**Affiliations:** Department of Life Sciences, Faculty of Science and Technology, The University of the West Indies, St. Augustine, Trinidad and Tobago, West Indies; Department of Pre-clinical Science, Faculty of Medical Sciences, The University of the West Indies, St. Augustine, Trinidad and Tobago, West Indies

**Keywords:** *Colletotrichum* spp, Entropy variability, Molecular phylogeny

## Abstract

The *Colletotrichum gloeosporioides* species complex is among the most destructive fungal plant pathogens in the world, however, identification of isolates of quarantine importance to the intra-specific level is confounded by a number of factors that affect phylogenetic reconstruction. Information bias and quality parameters were investigated to determine whether nucleotide sequence alignments and phylogenetic trees accurately reflect the genetic diversity and phylogenetic relatedness of individuals. Sequence exploration of GAPDH, ACT, TUB2 and ITS markers indicated that the query sequences had different patterns of nucleotide substitution but were without evidence of base substitution saturation. Regions of high entropy were much more dispersed in the ACT and GAPDH marker alignments than for the ITS and TUB2 markers. A discernible bimodal gap in the genetic distance frequency histograms was produced for the ACT and GAPDH markers which indicated successful separation of intra- and inter-specific sequences in the data set. Overall, analyses indicated clear differences in the ability of these markers to phylogenetically separate individuals to the intra-specific level which coincided with information bias.

## Introduction

*Colletotrichum gloeosporioides* is among the most pervasive and destructive fungal plant pathogens in the world (Sutton 1992; Cannon et al. 2008). *C. gloeosporioides* exists as a species complex (*Colletotrichum gloeosporioides sensu lato* (Weir et al. 2012) whose segregate taxa cannot be easily separated morphologically or phylogenetically and the approaches currently used to assign intra-specific ranking to these segregate taxa are still to be resolved and universally applied. Consequently, there is a preference among many for using the broad, group-species nomenclature rather than using names at the intra-specific level. However, this can cause confusion concerning the segregate taxa because there is overlap in morphological, biological and genetic variation at the intra-specific level. Correct identification of isolates of the *C. gloeosporioides sensu lato* species complex is important as some species pose quarantine risks some *Colletotrichum* species can infect multiple hosts.

Phylogenetic analysis of nucleotide sequences is one approach to achieving this level of taxonomic resolution. To date, low-level phylogenetic relationships in fungi have been inferred based on the sequence data for a number of nuclear and mitochondrial markers (Bridge et al., 2005; Vialle et al. 2009). However, the rationale behind the selection of these genes is not always apparent or reported – an important consideration since accurate tree reconstruction is critically dependent on the availability of suitably informative characters for phylogenetic analyses (Brito and Edwards 2009). Very few studies have been carried out to understand why some nuclear genes are better suited for phylogenetic reconstruction than others. Meaningful recovery of the phylogenetic hypothesis from a specific genetic marker may be complicated by factors such as a heterogeneous base composition (Lockhart et al. 1994), codon position saturation and transition/transversion rate bias (Phillips et al. 2004). Additionally, since some of these genes are duplicated in some fungal taxa there is the potential to infer phylogenetically inaccurate relationships (Ayliffe et al. 2001; Landvik et al. 2001; Tanabe et al. 2002; Tanabe et al. 2004 ; Fitzpatrick et al. 2006).

Multi-gene phylogenies are now commonly used to accurately define species in fungi and can be used to understand population history, demography and speciation (Taylor et al. 2000). However, different genes can evolve along different evolutionary trajectories which may result in tree inconsistencies (Brito and Edwards 2009; Aguileta et al. 2008; Townsend et al. 2008). In cases of very closely or distantly related taxa, certain aspects of the sequence data may contradict each other or carry insufficient or ambiguous information. Some researchers seek to address these irregularities and increase the robustness of phylogenetic inferences by increasing the sequence length of the marker used, thus increasing the amount of available data for inference (Bremer et al. 1999; van Oppen et al. 2001). This can be achieved artificially by concatenating the sequences of a number of different markers of distinct loci (Weir et al. 2012; Bremer et al. 1999; van Oppen et al. 2001; Slowinski and Lawson 2002). Consequently, before concatenation, it is important to screen sequences for homogeneity of base substitution and other congruence parameters. Ideally, the phylogenetic signal of each marker should be determined. Where there is incongruence, partitioning the data based on marker-specific substitution models is one approach to using concatenated sequences, however, combining sequences that possess a high phylogenetic signal with sequences that possess a low phylogenetic signal will not improve tree accuracy (Whelan et al. 2001; Posada and Crandall 2002; Egger et al. 2007). Posada and Crandall (2002), therefore, recommended the use of single gene trees for phylogenetic tree construction.

In an effort to regularize any reference to and application of the name *C. gloeosporioides*, Cannon et al. (2008) carried out epitypification work and presented epitype sequences for *C. gloeosporioides sensu stricto*. The identification of many species within the *C. gloeosporioides* species complex based on multi-gene analyses has indicated that these new species are phylogenetically distinct from the epitype strain of *C. gloeosporioides sensu stricto* (Rojas et al. 2010). Among the genetic markers used for species assignations were partial Actin (ACT), β-Tubulin (TUB2), Calmodulin (CAL), Glutamine synthetase (GS) and Glyceraldehyde 3-phosphate dehydrogenase (GAPDH) and the nuclear rDNA internally transcribed spacer (ITS) region (Weir et al. 2012; Cai et al. 2009). However, even based on an 8-gene multilocus data set, accurate assignment of isolates at the intra-specific level was not achieved in some cases and in others, there was considerable overlap (Weir et al. 2012). Weir et al. (2012) and Cai et al. (2009) concluded that within the *C. gloeosporioides* species complex GAPDH, CAL, and ACT genetic markers can be used as DNA barcodes but, ITS sequences do not facilitate intraspecies discrimination. Doyle et al. (2013) separated *C. gloeosporioides sensu lato* isolates infecting cranberry in the United States, however, when the Apn2/Mat genetic marker was compared with TUB2 and ITS markers (Silva et al. 2012) some isolates still had ambiguous phylogenetic placement based on separate gene tree assessment or as part of a concatenated data set.

Studies on information bias and quality parameters are especially important when controversial and poorly supported relationships are to be investigated as in the case of the *C. gloeosporioides* species complex. However, there is a consensus that (i) the use of multilocus genetic data sets is required to better resolve phylogenetic relationships within the species complex, (ii) there is a low level of phylogenetic resolution for some intra-specific members of the complex, and (iii) current markers may not allow phylogenetic assignment of all identified intra-specific members of this species complex (Cannon et al. 2008; Weir et al. 2012; Rojas et al. 2010; Cai et al. 2009; Silva et al. 2012).

It is hypothesized that information bias among currently used molecular markers is one reason why it is difficult to generate phylogenetic trees that accurately reflect the genetic diversity and phylogenetic relatedness of individuals of the *C. gloeosporioides* species complex and which will allow intra-specific demarcation among such individuals. The main objectives of this study were (i) to conduct pre-phylogenetic sequence data exploration of parameters that are known to affect phylogenetic signal and resolution; these included determining indices of disparity, base substitution bias, entropy variability and sequence heterogeneity among a select sequence data set: *C. gloeosporioides sensu lato* isolates associated with anthracnose of papaya in Trinidad were selected to be the test population, and (ii) to compare three algorithms with gene-specific models of evolution in creating gene trees for the identification and assignment of query isolates and epitypes to the intra-specific level based on multi-locus nucleotide sequence data. It is not the objective of this study to present new or novel isolates or to give definitive species assignment or phylogeny if the analyses applied do not allow it.

## Materials and methods

### DNA extraction, PCR, and sequencing

DNA was extracted using the E.Z.N.A. fungal DNA extraction kit® according to manufacturer’s instructions (Omega bio-tek Ltd., USA). Four markers were selected to generate sequences for quality analysis: partial Actin (ACT), partial β-Tubulin (TUB2), Glyceraldehyde-3-phosphate dehydrogenase (GAPDH) and internally transcribed spacer regions I and II (ITS) of the rDNA gene regions. The universal primer pair ITS4/5 was used in PCR to amplify the ITS region (496 bp) of the nuclear ITS1-5.8S-ITS2 rDNA (White et al. 1990); Bt2a/b primers allowed amplification of a 560 bp fragment of the TUB2 gene region (Glass and Donaldson 1995); GDF/GDR (Templeton et al. 1992) and ACT-512 F/ACT-783R (Carbone and Kohn 1999) primers were used to amplify 300 bp and 290 bp fragments, respectively. The standard calmodulin (CL1/CL2A) primers (O’Donnell et al. 2000) were not successful in generating any amplions for most isolates and this gene region was not subsequently used in the analysis. Each 25 μL PCR reaction contained 1 × PCR buffer; 1.5 mM MgCl_2_, 0.2 mM dNTP, 2.5 U Taq DNA Polymerase (Invitrogen by Life Technologies Co., USA) and 50 pmoles of each primer (Integrated DNA Technologies, USA). Thermal cycling conditions applied were: an initial denaturation of 5 min at 94°C followed by 35 cycles of 1 min at 94°C, 1 min at 55°C, 1 min at 72°C with a final extension of 5 min at 72°C. PCR products were sequenced directly (Amplicon Express, WA, USA).

### Data sets

Only holotype and epitype sequences of *C. gloeosporioides sensu stricto*, *C. asianum*, *C. fructicola*, *C. siamense* and *C. tropicale* were used in the study (Phoulivong et al., 2010; Cannon et al. 2012) (Table 1). These selected species have been analyzed in previous independent phylogenetic studies, and thus provides an objective reference in phylogenetic relationships.

**Table 1:** **Authentic sequences for accepted** ***Colletotrichum*** **species (Cannon et al.** 2008 **)**

Marker	GenBank Accession No.	Strain ^1^	Source	Species
GAPDH	FJ972582	CBS 95397	Culture from epitype	*Colletotrichum gloeosporioides sensu stricto*
GAPDH	FJ972577	MFU090228, ICMP 18581, CBS 130416	Culture from holotype	*Colletotrichum fructicola*
GAPDH	FJ972576	MFU 090233, ICMP 18580, CBS 130418	Culture from holotype	*Colletotrichum asianum*
GAPDH	FJ972575	MFU 090230, ICMP 18578, CBS 130417	Culture from holotype	*Colletotrichum siamense*
GAPDH	FJ972572	MFU 090230, ICMP 18578, CBS 130417	Culture from holotype	*Colletotrichum siamense*
GAPDH	FJ972571	MFU 090233, ICMP 18580, CBS 130418	Culture from holotype	*Colletotrichum asianum*
GAPDH	JX010078	ICMP 17673, ATCC 201874	Culture from holotype	*Colletotrichum tropicale*
GAPDH	GU888192	CBS:151.28	Culture from holotype	*Colletotrichum lindemuthianum*
ACT	FJ907430	CBS 95397	Culture from epitype	*Colletotrichum gloeosporioides sensu stricto*
ACT	FJ907426	MFU090228, ICMP 18581, CBS 130416	Culture from holotype	*Colletotrichum fructicola*
ACT	FJ907423	MFU 090230, ICMP 18578, CBS 130417	Culture from holotype	*Colletotrichum siamense*
ACT	FJ907420	MFU 090230, ICMP 18578, CBS 130417	Culture from holotype	*Colletotrichum siamense*
ACT	FJ903188	MFU 090233, ICMP 18580, CBS 130418	Culture from holotype	*Colletotrichum asianum*
ACT	JX009489	CBS 124949, ICMP18653	Culture from holotype	*Colletotrichum tropicale*
ACT	GU227898	CBS:151.28	Culture from holotype	*Colletotrichum lindemuthianum*
TUB	FJ907445	CBS 95397	Culture from epitype	*Colletotrichum gloeosporioides sensu stricto*
TUB	FJ907439	MFU 090233, ICMP 18580, CBS 130418	Culture from holotype	*Colletotrichum asianum*
TUB	FJ907435	MFU 090230, ICMP 18578, CBS 130417	Culture from holotype	*Colletotrichum siamense*
TUB	FJ907434	MFU 090233, ICMP 18580, CBS 130418	Culture from holotype	*Colletotrichum asianum*
TUB	JX010407	CBS 124949, ICMP18653	Culture from holotype	*Colletotrichum tropicale*
TUB	GU228094	CBS:151.28	Culture from holotype	*Colletotrichum lindemuthianum*
ITS	FJ972612	MFU 090233, ICMP 18580, CBS 130418	Culture from holotype	*Colletotrichum asianum*
ITS	FJ972611	MFU090228, ICMP 18581, CBS 130416	Culture from holotype	*Colletotrichum fructicola*
ITS	FJ972609	CBS 95397	Culture from epitype	*Colletotrichum gloeosporioides sensu stricto*
ITS	FJ972605	MFU 090233, ICMP 18580, CBS 130418	Culture from holotype	*Colletotrichum asianum*
ITS	FJ972604	MFU 090230, ICMP 18578, CBS 130417	Culture from holotype	*Colletotrichum siamense*
ITS	FJ972603	MFU090228, ICMP 18581, CBS 130416	Culture from holotype	*Colletotrichum fructicola*
ITS	JX010264	CBS 124949, ICMP18653	Culture from holotype	*Colletotrichum tropicale*
ITS	AY376534	STE-U 5297	Culture from ex-epitype	*Colletotrichum gloeosporioides sensu stricto*
ITS	EU371022	IMI 356878	Culture from epitype	*Colletotrichum gloeosporioides sensu stricto*
ITS	GU227800	CBS:151	Culture from holotype	*Colletotrichum lindemuthianum*

^1^Strain:

ATCC: American type culture collection, 10801 University Boulevard, Manassas, Virginia, USA.

CBS: Culture collection of the Centraalbureau voor Schimmelcultures, Fungal Biodiversity Centre, Utrecht, The Netherlands.

ICMP: International Collection of Microorganisms from Plants, Landcare Research, Auckland, New Zealand.

IMI: Culture collection of CABI Europe UK Centre, Egham, UK.

MFU: fungarium of Mae Fah Luang University, Thailand (cultures in BCC (BIOTEC Culture Collection, Thailand).

STE-U: Culture collection of the Department of Plant Pathology, University of Stellenbosch, South Africa.

**Figure 1:**
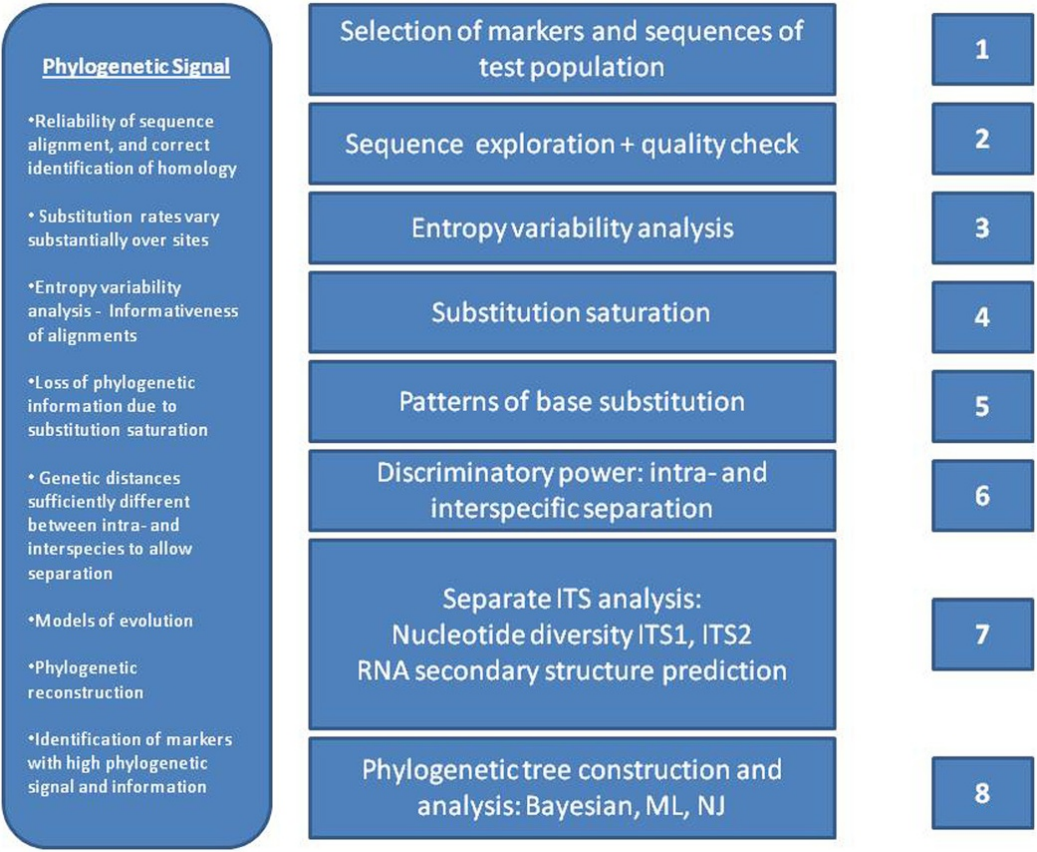
**Schematic of methods used in sequence exploration prior to phylogenetic reconstruction.**

In addition to the sequences derived in this study (Table 2), query sequences were also extracted from a previous study by Rampersad et al. (2013) and used in the final data set. Representative sequences were submitted to GenBank (GenBank: JQ218143, JQ218144, JQ218145, JQ218146). A total of 143 sequences for all markers was used in the final data set - 35 taxa for GAPDH; 34 taxa for ACT; 37 taxa for TUB2; 37 taxa for ITS. Alignments were carried out using the MAFFT server (http://mafft.cbrc.jp/alignment/server/) under default parameters. BioEdit software (http://www.mbio.ncsu.edu/BioEdit/bioedit.html). was used to edit the alignments of each marker data set. A schematic outlining the approach used to explore sequences is illustrated in Figure 1.

**Table 2:** **Isolate data**

Isolate	Field	Location	Cultivar	Year
PAW-Cg-110	Tableland	South Trinidad	Tainung No. 2-F1 hybrid	2011
PAW-Cg-111	Tableland	South Trinidad	Tainung No. 2-F1 hybrid	2011
PAW-Cg-112	Tableland	South Trinidad	Tainung No. 2-F1 hybrid	2011
PAW-Cg-113	Tableland	South Trinidad	Tainung No. 2-F1 hybrid	2011
PAW-Cg-114	Tableland	South Trinidad	Tainung No. 2-F1 hybrid	2011
PAW-Cg-115	Tableland	South Trinidad	Tainung No. 2-F1 hybrid	2011
PAW-Cg-116	Tableland	South Trinidad	Tainung No. 2-F1 hybrid	2011
PAW-Cg-118	Tableland	South Trinidad	Red Lady	2011
PAW-Cg-119	Tableland	South Trinidad	Red Lady	2011
PAW-Cg-120	Tableland	South Trinidad	Tainung No. 2-F1 hybrid	2011
PAW-Cg-121	Tableland	South Trinidad	Tainung No. 2-F1 hybrid	2011
PAW-Cg-122	Tableland	South Trinidad	Tainung No. 2-F1 hybrid	2011
PAW-Cg-5	Tableland	South Trinidad	Tainung No. 2-F1 hybrid	2011
PAW-Cg-6	Tableland	South Trinidad	Tainung No. 2-F1 hybrid	2011
PAW-Cg-7	Tableland	South Trinidad	Red Lady	2011
PAW-Cg-8	Barrackpore	South Trinidad	Red Lady	2011
PAW-Cg-102	Barrackpore	South Trinidad	Tainung No. 2-F1 hybrid	2011
PAW-Cg-103	Barrackpore	South Trinidad	Tainung No. 2-F1 hybrid	2011
PAW-Cg-104	Barrackpore	South Trinidad	Tainung No. 2-F1 hybrid	2011
PAW-Cg-105	Barrackpore	South Trinidad	Red Lady	2011
PAW-Cg-106	Santa Cruz	North Trinidad	Tainung No. 2-F1 hybrid	2011
PAW-Cg-107	Santa Cruz	North Trinidad	Tainung No. 2-F1 hybrid	2011
PAW-Cg-108	Santa Cruz	North Trinidad	Tainung No. 2-F1 hybrid	2011
PAW-Cg-109	Santa Cruz	North Trinidad	Red Lady	2011
PAW-Cg-9	Paramin	North Trinidad	Red Lady	2011
PAW-Cg-10	Paramin	North Trinidad	Red Lady	2011
PAW-Cg-41	Paramin	North Trinidad	Tainung No. 2-F1 hybrid	2011

### Net base composition bias disparity between sequences

Homogeneity tests can indicate different rates of substitution characteristic of certain lineages across different genes (Hedges and Kumar 2003). Several factors can influence substitution rates including the functionality of proteins and RNA, generation time, metabolic rate, population size, and life histories (Thomas et al. 2006; Smith and Donoghue 2008). Base composition for each gene according to test species was calculated in MEGA 5 (Tamura et al. 2011). The disparity index (ID) corresponds to the observed difference in base composition between two sequences compared to the compositional difference that would be expected under homogeneity of the evolutionary process. *I*_D_ equals 0 when the homogeneity assumption is satisfied. Values greater than 0 indicate that the larger differences in base composition bias than expected under homogeneity. Disparity Index (*I*_D_) per site was calculated for all sequence pairs. All positions containing gaps were omitted from the data set (complete deletion option). The significance of a given *I*_D_ value was then assessed using a Monte Carlo approach.

### Substitution saturation

Base substitution saturation decreases the amount of phylogenetic information contained in a sequence data set and can disrupt analysis involving deep phylogeny (Xia and Xie 2001). Homoplasy due to multiple substitutions was tested with the index of substitution saturation (ISS) (Xia et al. 2003a), which assumes a critical index of substitution saturation (ISSc) that defines a threshold for significant saturation in the data. The level of substitution saturation was assessed by using the substitution saturation test of the program package DAMBE v. 5.3.46 software package (Xia et al. 2003b) which determines an “index of substitution saturation”, based on the notion of entropy in information theory.

### Entropy variability analyses

Entropy is a measure of sequence variation and can be used to quantify the level of phylogenetic information available in sequence alignments (Shannon 1948; Krüger et al. 2012). A position has zero entropy (invariant alignment column) if it has the same character state in every sequence in the alignment, thereby making this nucleotide position phylogenetically uninformative. The maximum Shannon entropy is dependent on the number of discrete variable in the data set. We investigated the entropy landscape of each alignment by constructing entropy plots for each marker in BioEdit.

### Recombination detection

Multiple recombination detection methods were applied as implemented in RDP3 to detect recombination events in the aligned sequences for each gene. Using RDP3, four methods were applied: RDP (Martin and Rybicki 2000), GENECONV (Padidam et al. 1999), MaxChi (Maynard 1992) and 3Seq (Boni et al. 2007). Sequences were treated as linear and the threshold *P*-value was set at 0.05, using Bonferroni correction. All other settings were set to default. Methods like GENECONV and MAXCHI do not include phylogenetic information in their recombination detection algorithms and phyogenetic trees generated did not include any recombination event information.

### Differentiation between intra- and interspecific sequences

The genetic distances were calculated under the Kimura 2 parameter (K2P) model using MEGA 5 for all sequences of the data set for each marker. Frequency histograms were generated to examine the intra- and possible interspecific variation among genetic distances. Using the genetic distances from pair-wise comparisons, the percentage comparisons with genetic distances greater than 5% are reported. These cut-off values are arbitrary but may indicate less inclusive clades. The distribution and size of the modes depend on the degree and range of sequence divergence and the number of intra-specific and interspecific taxa in the data set.

### ITS sequence analysis

Errors can occur during amplification when two different sequences are used as template DNA. The PCR product may include a combination of these original sequences (Jumpponen 2011). Such chimeric sequences may be misinterpreted as novel which can artificially inflate estimates of diversity and interfere with phylogenetic inference and species discrimination if undetected (Hugenholtz and Huber 2003). ITS sequences were checked for putative chimeras using the UNITE PlutoF Chimera checker (Edgar et al. 2011) and the Chimera Test developed in the Fungal Metagenomics Project at the University of Alaska (https://biotech.inbre.alaska.edu/fungal_portal/?program=chimera_test).

### Verifying ITS sequence validity

Alignments were carried out using the online version of the sequence alignment program MAFFT version 6 ((http://mafft.cbrc.jp/alignment/server/) to ensure that the 5.8S region of the ITS array forms an anchor region in the middle of the alignment. This check will determine if the ITS sequences were composed of stochastic, artifactual nucleotide data.

### Phylogenetic hypotheses

Multiple sequence alignments of each gene were made and manually adjusted where necessary with BioEdit. Individual gene trees were reconstructed using the Bayesian Markov chain Monte Carlo (MCMC) approach as implemented in Mr. Bayes 3.2.1 (Huelsenbeck and Ronquist 2001), and Neighbour joining (NJ) and Maximum Likelihood (ML) algorithms implemented in PAUP* version 4.0b8 (D. L. Swofford, Sinauer Associates, Sunderland, MA, Swofford 2003). For each of the markers, tree topologies were compared and evaluated for disagreement.

The Mr. Bayes analyses were performed under the GTR + Γ + I model. To ensure accuracy, four parallel runs were carried out with one cold and three heated MCMC chains per run for 1,100,000 generations; trees were sampled every 200 generations and the first 100,000 trees were discarded as these represented the trees of the burn-in phase of the analysis. Convergence was assessed by examining the stationarity of the ln-likelihood and effective sample size which was >500 for all analyses. The remaining trees were summarized as a Maximum Clade Credibility (MCC) tree (i.e. tree topology with the highest clade posterior probabilities across all nodes).

Bootstrap-supported (1,000 replicates) maximum likelihood (ML) and neighbour-joining (NJ) trees were estimated under their respective best-fit models of nucleotide substitution as determined using ModelTest v.3.7 (Posada 2008) with corrected Akaike information criteria (AICc). The models used were TUB2: HKY85; ACT: F81; ITS: GTR and GAPDH: GTR. Separate sequences of the ITS1 and ITS2 loci were used in a second and third data set to generate Bayesian trees under the GTR + Γ + I model, and ML and NJ phylogenetic trees based on the best-fit evolutionary model which were HKY85 for ITS1 and GTR for ITS2.

We included sequence data from representative epitype isolates which were selected as authentic cultures by the CBS (Centraalbureau voor Schimmelcultures institute, Utretcht, Netherlands), IMI (CABI Europe – UK, Bakeham Lane, Egham, Surrey, UK) and/or as cultures listed as authentic by Cannon et al. (2012) (Table 1). We also sought to reduce the level of incongruence between individual gene trees by using the approach of conditional editing of sequences (Salichos and Rokas 2013) where (i) sites containing gaps and any aberrant sequences that produced a “bad” alignment were removed from the data set, (ii) quickly evolving taxa that are attracted by the distant outgroup or taxa that resulted in long branch attraction were removed, (iii) a limited number of taxa in each gene tree (query and epitype), (iv) only established or known ex-epitype and epitype sequences and (v) genes that recover specific internodes and where species placement has proven consistent among independent studies were used. FigTree was used to annotate trees and export graphics.

## Results

MAFFT analysis revealed no evidence of chimeric sequences present in the ITS data set and indicated good quality ITS sequences with no artifactual nucleotide data or pseudogenes. When the separate ITS loci were analyzed, it was found that the ITS1 region was more variable than the ITS2 region (Pi _ITS1_ = 0.01068; Pi _ITS2_ = 0.00390).

In this study, tests of the homogeneity of substitution patterns in query sequences indicated that isolates had different patterns of nucleotide substitution for ACT and TUB2 markers (Table 3). For the ITS sequence data, all isolates had *P*-values lower than 0.05; for the TUB2 sequence data set (*N* =29) 12 isolates had *P*-values lower than 0.05; for the GAPDH sequence data set (*N* =27) 9 isolates had *P*-values lower than 0.05. None of the isolates had a *P*-value lower than 0.05 for the ACT data set (*N* =27).

**Table 3:** **Tests of homogeneity of base substitution patterns**

Marker	Number of positions in final data set	Number of isolates ^1^	Isolate ID
ACT	158	3	PAW-Cg-118, PAW-Cg-107 and PAW-Cg-108
GAPDH	164	0	…
ITS	481	0	…
TUB2	287	11	PAW-Cg-9, PAW-Cg-111, PAW-Cg-112, PAW-Cg-114, PAW-Cg-116, PAW-Cg-119, PAW-Cg-121, PAW-Cg-8, PAW-Cg-9 and PAW-Cg-41

^1^A Monte Carlo test (1000 replicates) was used to estimate the *P*-values (*P* ≤0.05); the number and identity of isolates with a *P*-value smaller than 0.05. All positions containing gaps and missing data were eliminated from the data set (complete deletion option).

### Base substitution saturation

**Figure 2:**
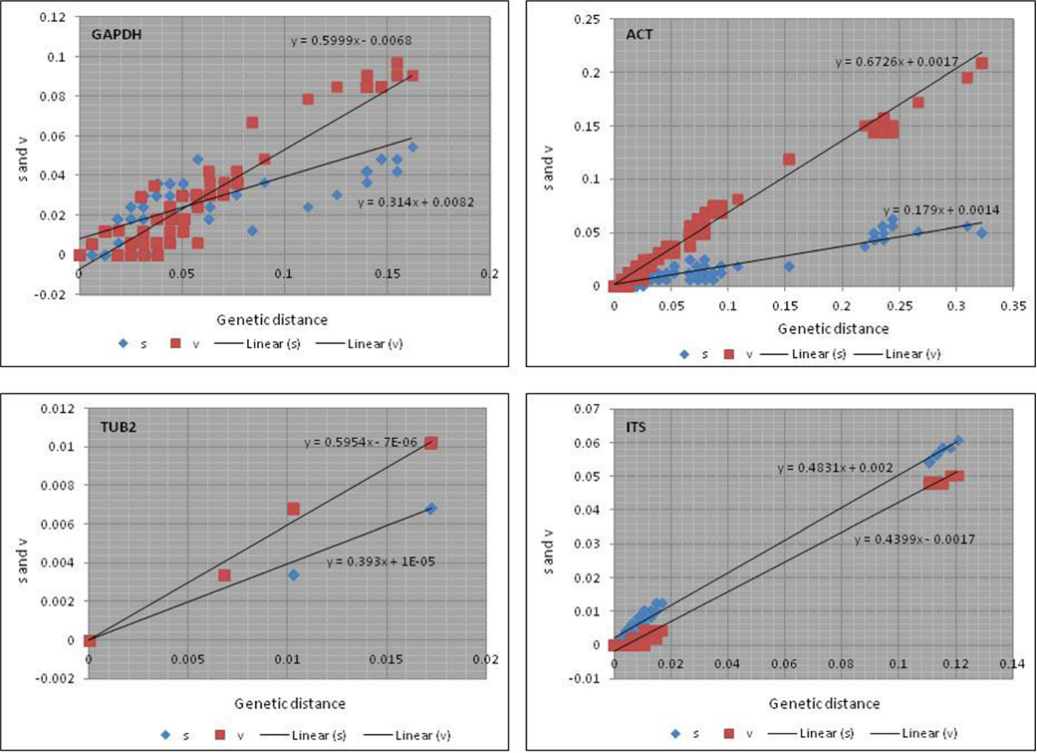
**DAMBE base substitution saturation plots for GAPDH, ACT, TUB2 and ITS sequence data sets.**

Tests to determine base substitution saturation were conducted for all markers separately, for combined ITS array data set, and for the stem and loop regions of ITS1 and ITS2 regions separately (Table 4). Results indicated that for each data set, ISS values were consistently lower than the ISSc values which indicated little saturation in base substitution. The test also revealed little difference between paired (stem) and unpaired positions (loops). In the stem and loop regions of all relevant ITS data sets, the ISS value was always lower significantly (*P* <0.0001) than the observed ISSc. The complete results of the saturation tests are summarised in Table 3. The findings of the saturation test suggested that there was no need to partition the stem and loop regions of the ITS1 and ITS2 data sets for phylogenetic analyses as these regions had little saturation.Estimation of saturation can also be determined based on the slope of the regression line in the saturation plot; data sets without any saturation have a slope =1, data sets with high saturation have a slope =0. Generally, base saturation by transitions was higher than base saturation by transversions except for the ITS markers which had near equivalent saturation for both transition- and transversion-type base substitution (Figure 2). Slopes for transversion-type and transition-type base substitutions were closer to 1 for ACT, GAPDH, TUB2 and ITS, in the order of least to most saturated.

**Table 4:** **Comparison of the index of substitution saturation (ISS) with the critical index of substitution saturation (ISSc) that defines a threshold for significant saturation in the data**

Marker	Iss	Issc ^1^	T	DF	***P***
**ACT**	0.0609	0.6383	37.0781	155.0000	0.0000
**GAPDH**	0.0454	0.6162	36.7266	163.0000	0.0000
**ITS**	0.0167	0.7042	146.3186	477.0000	0.0000
**TUB2**	0.0089	0.6636	87.1685	292.0000	0.0000
**ITS1-Loops**	0.1665	0.9113	13.6810	50.0000	0.0000
**ITS2-Loops**	0.0425	0.8548	34.1213	59.0000	0.0000
**ITS1-Stem**	0.0390	0.4671	19.9356	102.0000	0.0000
**ITS2-Stem**	0.0481	0.4980	16.5953	96.0000	0.0000

^1^Only the symmetrical tree topology is presented.

### Entropy variability analysis

**Figure 3:**
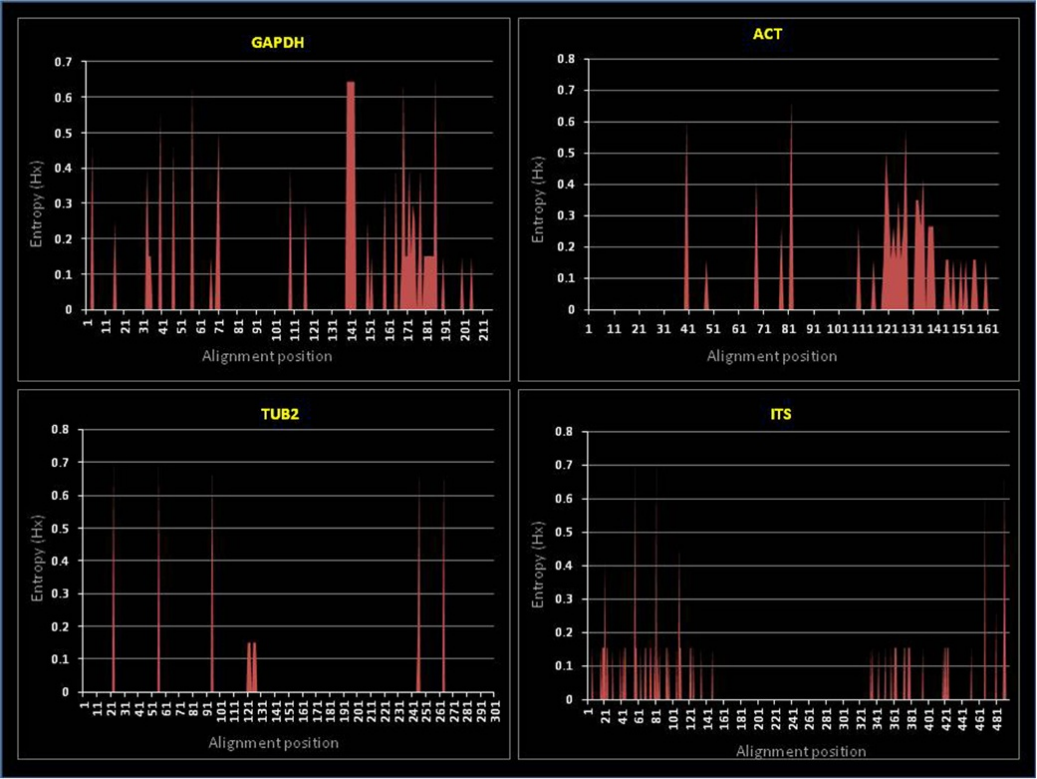
**Entropy plots showing the relative entropy distributions within the GAPDH, ACT, TUB2 and ITS sequence alignment data sets.**

Shannon entropy distributions within the GAPDH, ACT, TUB2 and ITS DNA sequence alignments were examined. Regions of high entropy were the most dispersed in the GAPDH marker alignment. Entropy plots (Figure 3) revealed only two high entropy clusters extending greater than eight sites. GAPDH had the highest number of peaks over 0.25 (18 peaks with three over 0.5 maximum), followed by ACT with eight peaks over 0.25 (only one over 0.5 maximum), ITS had seven peaks (three over 0.5 maximum) and TUB2 had five peaks (all of which were over a 0.5 maximum. Similar results were obtained for the translated amino acid alignment for reading frames 1, 2 and 3 of the TUB2 gene, data not shown). With respect to the ACT marker, the plot indicated a distinct cluster of sites towards the end of the alignment (alignment position 110 to 141) with entropy values greater than 0.25. For the ITS alignments, most of the entropy values occurred in two distinct clusters and which corresponded to the more variable ITS1 and ITS2 loci that flank the 5.8S region.

### Recombination detection

Aligned sequences of each gene were analyzed with recombination detection software, using the three methods of analyses i.e. RDP, GENECONV and MaxChi, with the aim of identifying possible recombinant isolates and to locate the recombined regions in these recombinant sequences. Recombination was detected in the aligned ACT gene sequences. No significant recombination events above the *P*-value threshold of 0.05were detected for MAXCHI analyses. 3Seq detected 2 isolates with significant (*P* <0.05) recombination events; PAW-Cg-107 and PAW-Cg-41; however, as none of the other methods detected these sequences, it is not certain whether these isolates were actual recombinants. There was no evidence of recombination in any of the other gene sequences.

### Intra- and interspecific differentiation

**Figure 4:**
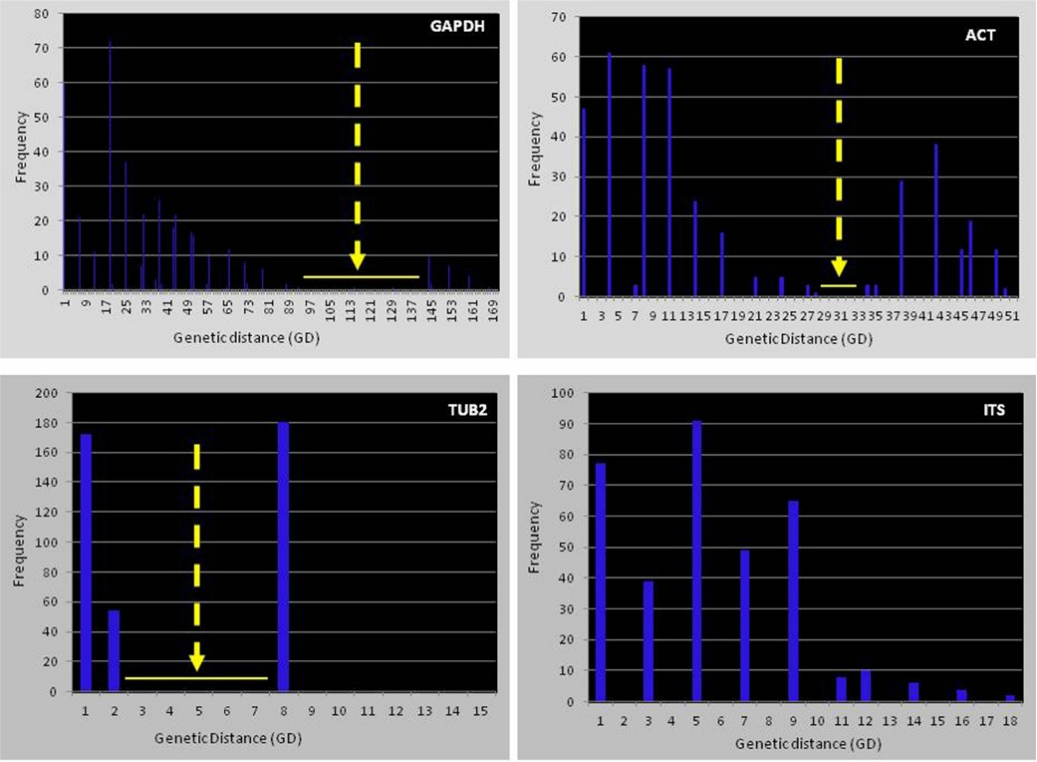
**Frequency histograms to examine the intra- and inter-specific variation for each marker based on pair-wise genetic distances.** The arrow indicates the frequency gap that separates intra-specific sequences.

The genetic distances for all sequences of the isolates in the data set for each marker were calculated under the Kimura 2-parameter (K2P) model using MEGA5 (Table 5). Frequency histograms were generated to examine the intra- and possible inter-specific variation based on pair-wise genetic distances. Summary statistics for genetic distance data are presented in Table 3. There is an apparent difference in the ability of a given markers to distinguish between intra-specific and interspecific sequences according to marker. The ACT and GAPDH markers were able to produce a discernible modal gap in the frequency histograms which indicate separation of intra- and inter-specific isolates (Figure 4). There is some uncertainty for TUB2 whether a true gap exists because the separation is not an easily discernible bimodal distribution as for the other markers. No discernible gap was observed for the ITS marker.

**Table 5:** **Comparison of genetic distance statistics for each marker**

Marker	Max GD ^1^	Min GD	Med GD	Std Dev GD	≥5% ^2^
**GAPDH**	0.167	0.000	0.085	0.0495	97
**TUB2**	0.018	0.000	0.010	0.0093	0
**ACT**	0.098	0.000	0.052	0.0323	122
**ITS**	0.057	0.000	0.007	0.0263	0

^1^Summary statistics of genetic distances calculated using the Kimura 2-paramter (K2P) model and include: Maximum genetic distance (Max GD), Minimum genetic distance (Min GD), Median genetic distance (Med GD) with standard deviation (Std Dev GD) and the number of pair-wise comparisons of genetic distances greater than or equal to 5% (≥5%).

^2^The number of pair-wise comparisons of genetic distances greater than or equal to 2% (≥2%) was also determined to be zero for ITS.

### Phylogenetic analysis

**Figure 5:**
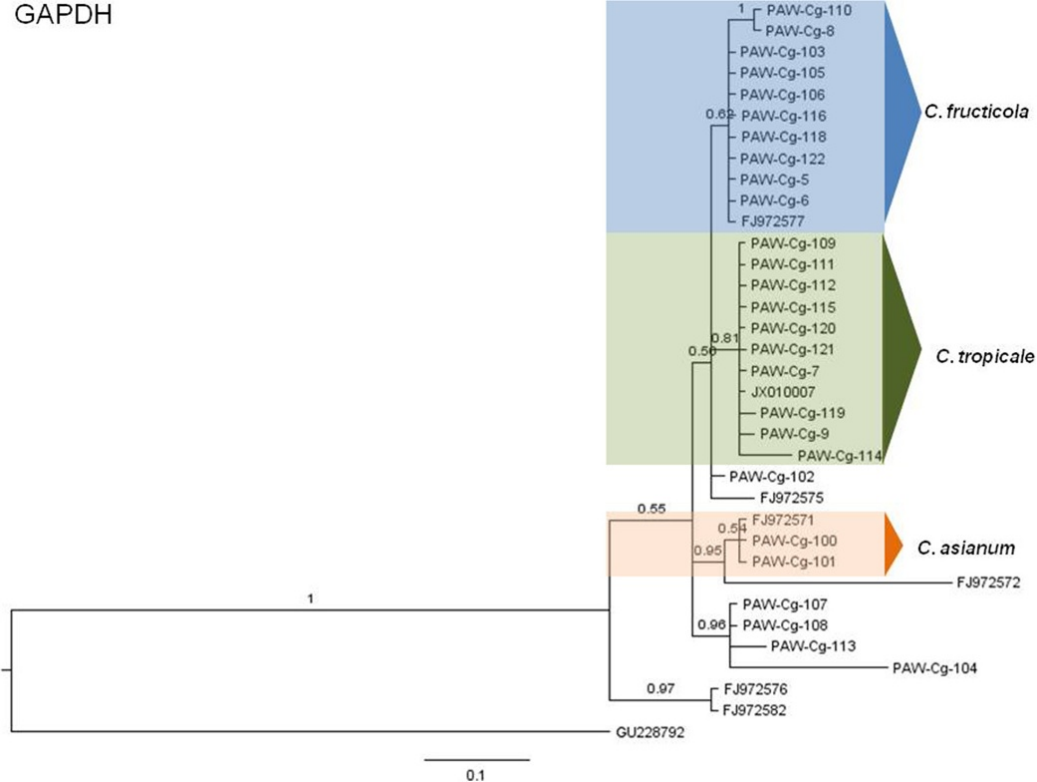
**Bayesian MCC phylogenetic tree for the GAPDH marker.** Numbers above branches are clade credibility scores.

**Figure 6:**
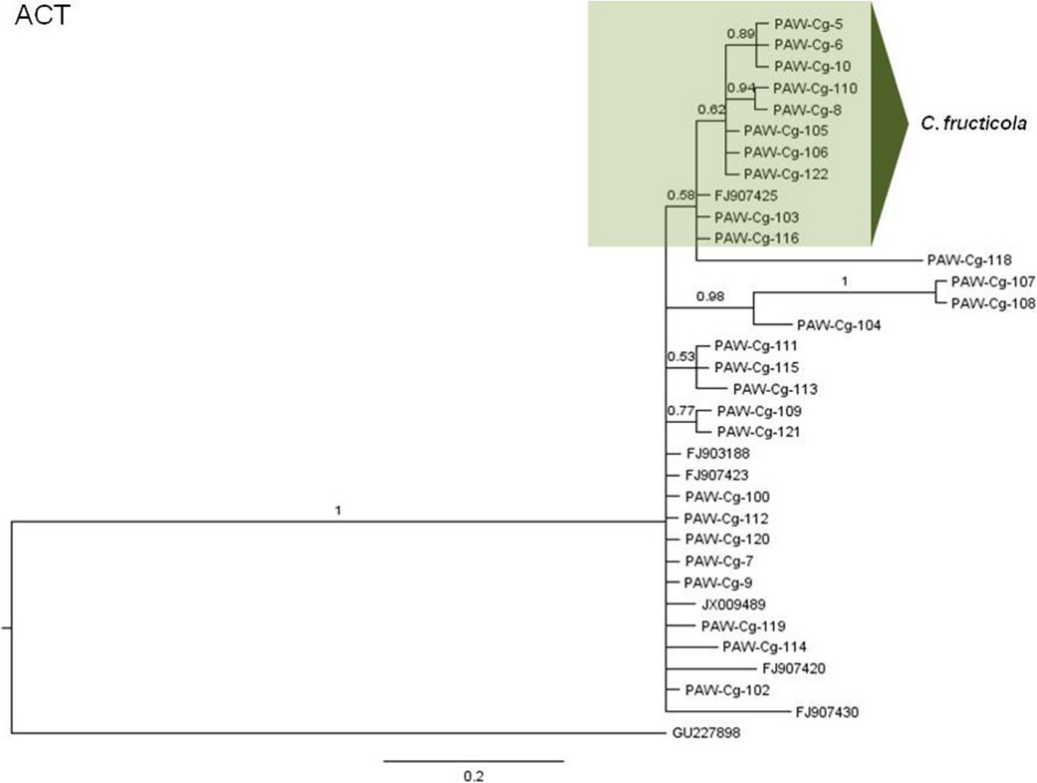
**Bayesian MCC phylogenetic tree for the ACT marker.** Numbers above branches are clade credibility scores.

**Figure 7:**
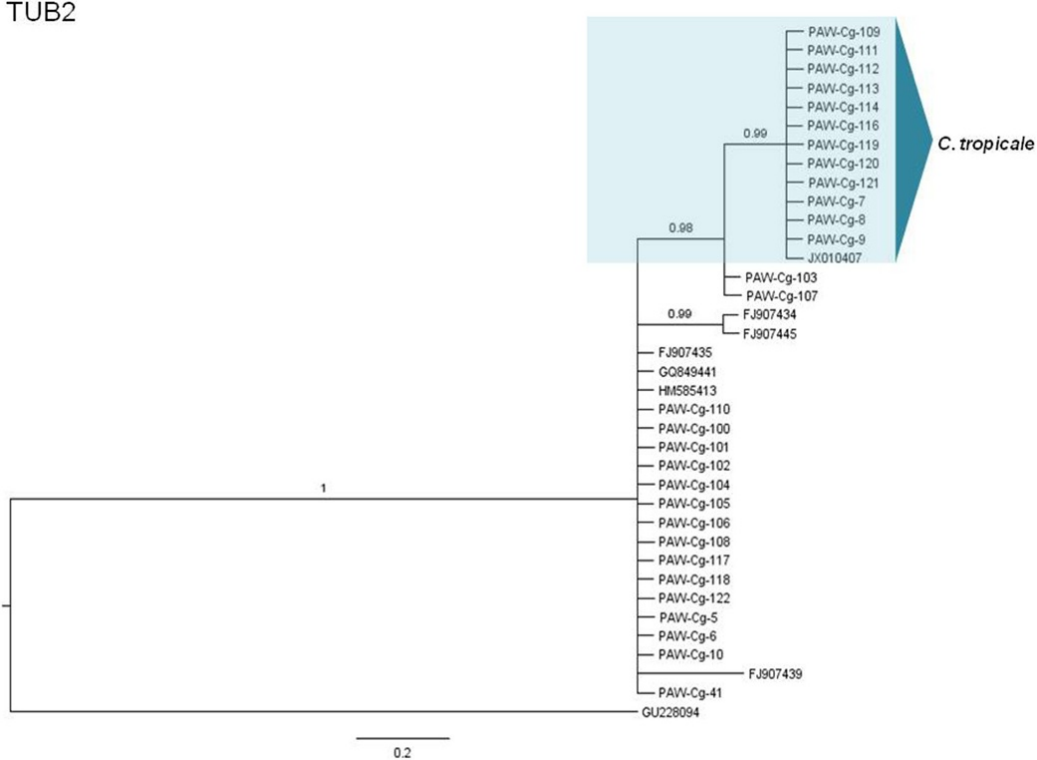
**Bayesian MCC phylogenetic tree for the TUB2 marker.** Numbers above branches are clade credibility scores.

**Figure 8:**
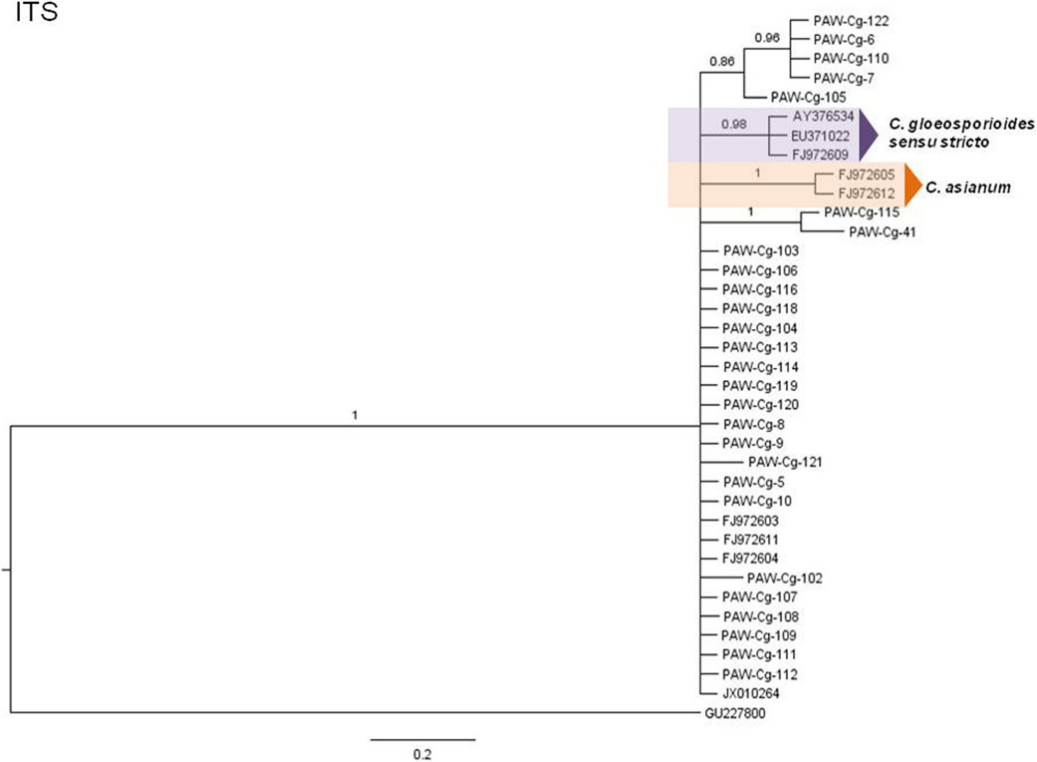
**Bayesian MCC phylogenetic tree for the ITS marker (entire ITS1-5.8S-ITS2 region).** Numbers above branches are clade credibility scores.

**Figure 9:**
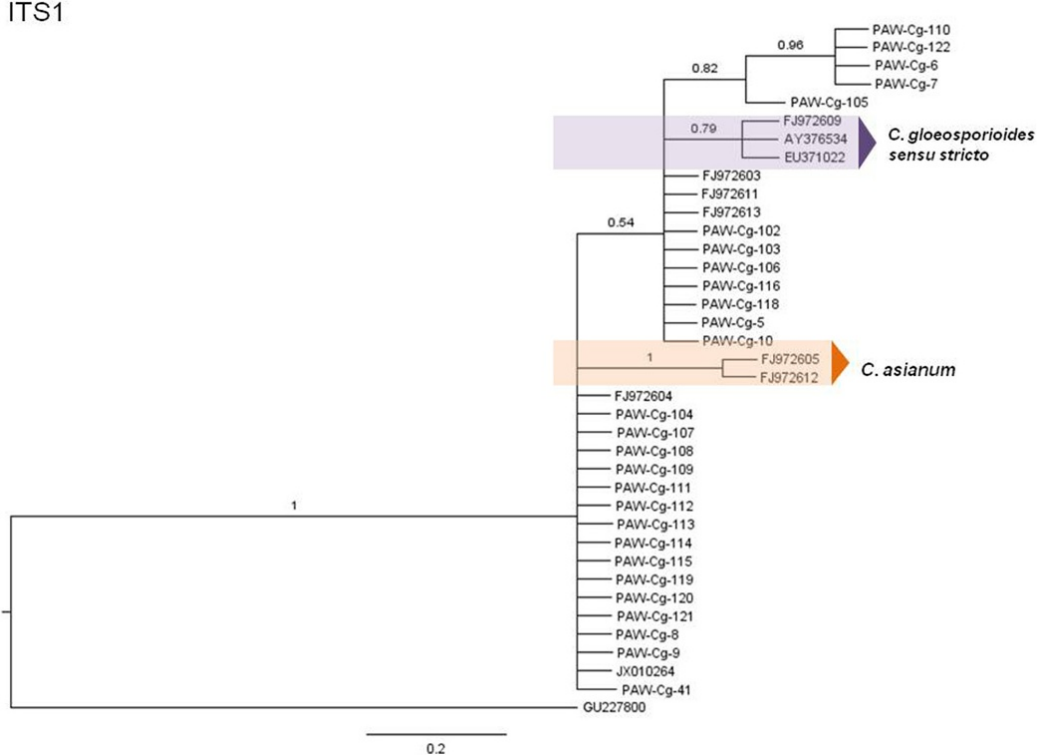
**Bayesian MCC phylogenetic tree for the ITS1 marker.** Numbers above branches are clade credibility scores.

**Figure 10:**
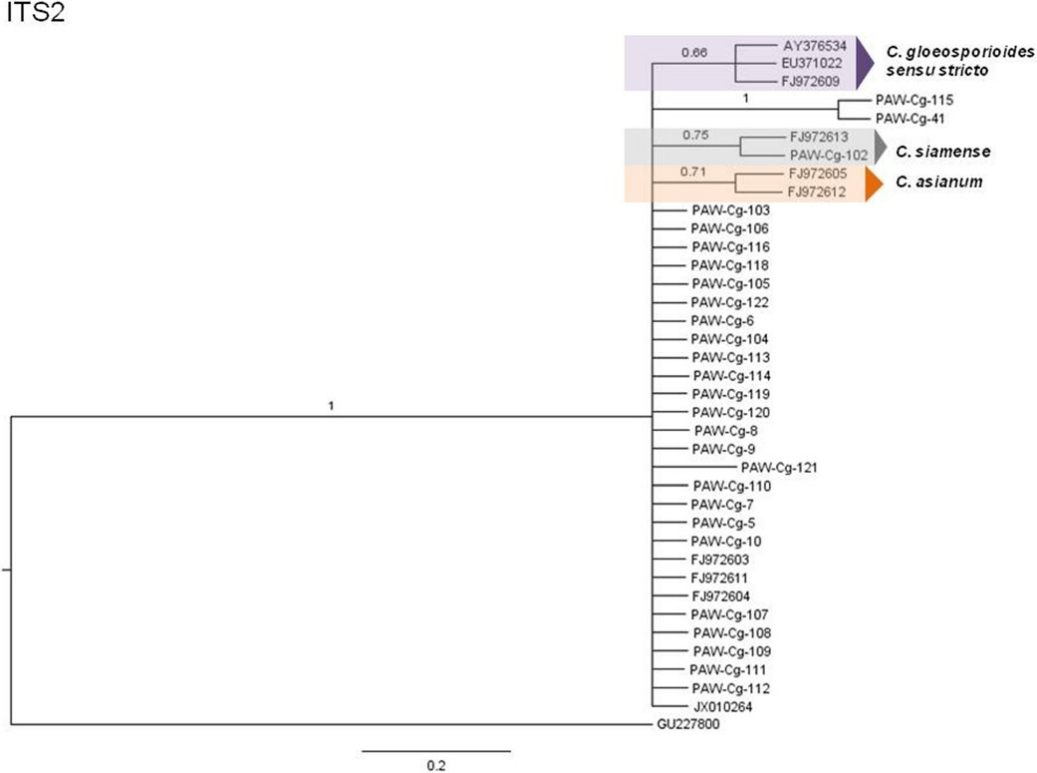
**Bayesian MCC phylogenetic tree for the ITS2 marker.** Numbers above branches are clade credibility scores.

The phylogenetic hypotheses for the Bayesian MCC trees are described here (Figures 5, 6, 7, 8, 9 and 10). Individual loci were analyzed separately to assess the topological congruence among the different sequence data sets. The ability of each data set to efficiently resolve terminal lineages with robust branch support for relationships within the *C. gloeosporioides sensu lato* species complex was determined. The topologies for the Bayesian MCC, ML and NJ trees were compared for each marker and were found to be congruent.

The GAPDH Bayesian MCC tree (Figure 5) also revealed a distinct *C. fructicola* clade with one subclade consisting of sister taxa PAW-Cg-110 and PAW-Cg-8 similar to the ACT tree. *C. tropicale* formed a separate clade along with nine isolates but forming a polytomic phylogeny. The *C. asianum* clade consisted of PAW-Cg100 and PAW-Cg-101 with one epitype sequence. For the ACT and GAPDH analyses, four isolates (PAW-Cg-104, PAW-Cg-107, PAW-Cg-108, PAW-Cg-113) were commonly positioned in both trees.

For the ACT tree (Figure 6), there was a distinct *C. fructicola* clade but with only moderate clade support, and with two subclades. *C. siamense*, *C. gloeosporioides sensu stricto*, *C. asianum* and *C. tropicale* epitype sequences formed a polytomic phylogeny with seven isolates in the data set. PAW-Cg-107 and PAW-Cg-108 appeared to be sister taxa and formed a separate strongly-supported clade with PAW-Cg-104. Similarly PAW-Cg-109 and PAW-Cg-121 also appeared to be sister taxa forming a well-supported clade. PAW-Cg-118, PAW-Cg-107 and PAW-Cg-108 appear to be faster evolving or more genetically divergent isolates than the others given the comparatively longer branch lengths.

The TUB2 Bayesian MCC tree (Figure 7) indicated a strongly-supported separate *C. tropicale* clade, consisting of 12 isolates which was identical to the *C. tropicale* clade of the GAPDH tree. There may be an apparent *C. siamense* clade consisting of 15 isolates but these formed a basal polytomic phylogeny.

The ITS Bayesian MCC tree (Figure 8) displayed with a distinct *C. gloeosporioides sensu stricto* clade consisting only of the three epitype sequences used in the data set. Similarly, a distinct *C. asianum* clade was discernible with strong branch support. *C. siamense*, *C. fructicola* and *C. tropicale* epitype sequences formed a basal polytomic phylogeny with the majority of the isolates in the data set.

The separate ITS1 and ITS2 loci were considered. For the ITS1 region tree (Figures 9 and 10), *C. fructicola* and *C. tropicale* formed a polytomic phylogeny with the majority of the isolates. *C. siamense* formed a separate polytomic clade with seven isolates; the three *C. gloeosporioides sensu stricto* and the *C. asianum* epitypes formed two separate clades with higher branch support than for the ITS2 region tree. For the ITS2 region tree, *C. fructicola* and *C. tropicale* together with the majority of isolates formed a polytomic phylogeny; the three *C. gloeosporioides sensu stricto* epitypes, one *C. siamense* epitype and one query isolate and the two *C. asianum* epitypes formed separate moderately-supported clades.

Overall, analyses indicated that the ACT marker resolved only the *C. fructicola* clade; the GAPDH marker resolved the *C. fructicola*, *C. tropicale* and *C. asianum* clades; the TUB2 marker resolved the *C. tropicale* and *C. siamense* clades; and the ITS marker (entire) resolved the *C. gloeosporioides sensu stricto* and the *C. asianum* clades; the ITS1 region resolved the *C. gloeosporioides sensu stricto* and the *C. asianum* clades and the ITS2 region resolved the *C. gloeosporioides sensu stricto, C. siamense and* the *C. asianum* clades.

## Discussion

It was hypothesized that information bias among currently used molecular markers is one reason why it is difficult to generate phylogenetic trees that accurately reflect the genetic diversity and phylogenetic relatedness of individuals of the *C. gloeosporioides* species complex and as such intra-specific demarcation among such individuals remain a challenge. Tests that detect information bias were conducted.

The results indicated differences in saturation levels, entropy and heterogeneity among the genetic markers tested. Comparisons with phylogenetic analyses demonstrate an apparent correlation between the detected information bias and resolution of phylogeny. It is, therefore, important to conduct such tests in support of any proposed phylogenetic hypothesis.

Our data also showed a distinct difference in the number of isolates in the test population achieving phylogenetic placement which was dependent on the marker. None of the isolates in the test population were resolved phylogenetically based on the entire ITS, or the separate ITS1 or ITS2 region. However, differences in the ability of the separate ITS loci to separate epitype sequences were detected. One explanation for the branch support difference between the ITS1 and ITS2 region trees with respect to separating *C. gloeosporioides sensu stricto* from *C. siamense* can be found in the level of nucleotide differences (Pi) between the two loci.

GAPDH was able to position the greatest number of isolates in the test population, it had the highest entropy distribution, it was the second marker with the least saturation and it was characterized by a distinct frequency gap that correlated to the largest genetic distance compared to the other markers. Importantly, the data also suggested that ACT and TUB2 served to confirm the GAPDH phylogeny of the isolates in the test population for the *C. fructicola* and *C. tropicale* clades, respectively. Weir et al. (2012) and Rojas et al. (2010) also concluded that these markers were able to resolve some species but not others, and many species could not be discriminated using a single gene.

The phylogenetic relationships of closely related taxa that resulted from rapid radiation over a very short period time are difficult to define as is the case for *C. gloeosporioides* species complex. It is possible that some member species in the *C. gloeosporioides* species complex may have been pre-maturely given new species designations (i.e. before they developed independent evolutionary and phylogenetic routes) which would help to explain the low level of phylogenetic signal and poor or incomplete resolution. Hoelzer and Meinick (1994) purported that regardless of the model and concepts used to define and identify a species, geographically isolated and genetically divergent subspecies will have complicated patterns of gene flow with other subspecies over evolutionary time and will emerge prior to actual “speciation”. Examination of a greater number of loci in the descendants will still lead to an inaccurate estimation of the pattern of bifurcations in the phylogenetic trees even though the bifurcations may have robust statistical support (Rokas and Carroll 2006).

As with any approach that imposes structure on the data (Hoelzer and Meinick 1994; Whitfield and Lockhart 2007) bifurcations result from an imposition of the tree-building method and necessarily reality (Chan and Moore 2005). If there is a real dichotomous structure in the data, the bifurcations will be apparent and the unresolved nodes will occur usually at or near the terminal branches, as seen in the trees generated in this study. “Hard” polytomies in phylogenetic reconstructions, as seen in this study and others (Weir et al. 2012; Kliman et al. 2000; Takahashi et al. 2001; Coyne et al. 2004) should be viewed as real multifurcations or multiple, simultaneous divergence events, rather than incomplete or unsuccessful resolution of evolutionary history (Maddison 1989). Polytomies may represent the most accurate possible representation of historical relationships among the taxa under consideration (Rokas and Carroll 2006; Felsenstein 1985; Grafen 1989).

The low level of genetic diversity within this species complex as reflected by the short branch lengths suggests that the species recognised within the *C. gloeosporioides* complex are very recently evolved (Cannon et al. 2012; Silva et al. 2012), therefore, it is likely that even with potentially more informative genes such as ApMAT and Apn25L (Silva et al. 2012) the low levels of genetic divergence across the *C. gloeosporioides* complex may continue to test the limits of phylogenetic resolution. If speciation events have only recently occurred among related taxa, the amount of informative phylogenetic data is low and the phylogenetic signal is small, leading to short internal tree branches or polytomies that are difficult to resolve (Saitou and Nei 1986; Philippe et al. 1994). Moreover, increasing the number of gene sequences in an attempt to solve a difficult phylogenetic question may not necessarily be the best approach if the data set has been generated from markers with low and high levels of phylogenetic information; the low phylogenetic signal may become dominant and yield inconsistent, yet highly supported, phylogenetic trees (Jeffroy et al. 2006).

## Conclusion

Sequence data exploration of parameters known to affect phylogenetic signal and resolution is important to understanding the phylogenetic placement of individuals. The findings of this study demonstrated that these parameters explained the ability of different markers to adequately identify individuals at the intra-specific level. This data is important to verify whether nucleotide sequence alignments and phylogenetic trees accurately reflect the genetic diversity and phylogenetic relatedness of individuals and to establish if any of the markers and taxa included in the data set show a strong departure from basic assumptions in phylogenetic tree reconstruction. The findings of this work should be considered in the context of (i) the existence of information bias among the genetic markers used for generating the phylogenetic hypothesis and (ii) time of sequence divergence of isolates at a rate that appears to be faster than the rate of speciation of these isolates or conversely, divergence of certain gene sequences may occur at a rate that is slower than the time for speciation.
